# Daytime sleepiness and sleep quality in Charcot–Marie–Tooth disease

**DOI:** 10.1007/s00415-023-11911-y

**Published:** 2023-08-04

**Authors:** Marta Bellofatto, Luca Gentile, Alessandro Bertini, Irene Tramacere, Fiore Manganelli, Gian Maria Fabrizi, Angelo Schenone, Lucio Santoro, Tiziana Cavallaro, Marina Grandis, Stefano C. Previtali, Marina Scarlato, Isabella Allegri, Luca Padua, Costanza Pazzaglia, Flavio Villani, Eleonora Cavalca, Paola Saveri, Aldo Quattrone, Paola Valentino, Stefano Tozza, Massimo Russo, Anna Mazzeo, Giuseppe Vita, Sylvie Piacentini, Giuseppe Didato, Chiara Pisciotta, Davide Pareyson, for the Italian C. M. T. Network, Giulia Schirinzi, Maria Montesano, Sara Nuzzo, Francesca Oggiano, Daniela Calabrese, Chiara Gemelli, Yuri Falzone, Emanuele Spina, Maria Longo, Giuseppe Occhipinti, Giacomo Iabichella, Stefania Barone

**Affiliations:** 1 SC Malattie Neurologiche Rare, Dipartimento di Neuroscienze Cliniche, Fondazione IRCCS Istituto Neurologico Carlo Besta, Via Celoria 11, 20133 Milan, Italy; 2 Unità di Neurologia e Malattie Neuromuscolari, Dipartimento di Medicina Clinica e Sperimentale, Università di Messina, 98124 Messina, Italy; 3 Dipartimento Gestionale di Ricerca e Sviluppo Clinico, Direzione Scientifica, Fondazione IRCCS Istituto Neurologico Carlo Besta, 20133 Milan, Italy; 4 Dipartimento di Neuroscienze, Scienze Riproduttive ed Odontostomatologiche, Università Federico II di Napoli, 80131 Naples, Italy; 5 Dipartimento di Neuroscienze, Biomedicina e Movimento, Università di Verona, 37126 Verona, Italy; 6 Dipartimento di Neuroscienze, Riabilitazione, Oftalmologia, Genetica e Scienze Materno-Infantili, Università di Genova, 16132 Genoa, Italy; 7 IRCCS Ospedale Policlinico San Martino, 16132 Genoa, Italy; 8 INSPE and Division of Neuroscience, IRCCS Ospedale San Raffaele, 20132 Milan, Italy; 9 U.O. Neurologia, A.O. di Parma, 42126 Parma, Italy; 10 Università Cattolica del Sacro Cuore, 00168 Rome, Italy; 11 Fondazione Policlinico Universitario A. Gemelli IRCCS, 00168 Rome, Italy; 12 Unità di U.O. Neurofisiopatologia, IRCCS Ospedale Policlinico San Martino, 16132 Genoa, Italy; 13 Dipartimento di Scienze Mediche, Università Magna Grecia, 88100 Catanzaro, Italy; 14 Unità di Neuropsicologia, Dipartimento di Neuroscienze Cliniche, Fondazione IRCCS Istituto Neurologico Carlo Besta di Milano, 20133 Milan, Italy; 15 Unità di Epilettologia Clinica e Sperimentale, Dipartimento di Neuroscienze Cliniche, Fondazione IRCCS Istituto Neurologico Carlo Besta, 20133 Milan, Italy

**Keywords:** Hereditary motor and sensory neuropathies, Charcot–Marie–Tooth disease, Sleep, Sleepiness, Fatigue, Depression

## Abstract

**Background:**

Sleep abnormalities have been reported in Charcot–Marie–Tooth disease (CMT), but data are scanty. We investigated their presence and correlation in a large CMT patients’ series.

**Methods:**

Epworth Sleepiness Scale (ESS) and Pittsburgh Sleep Quality Index (PSQI) were administered to CMT patients of the Italian registry and controls. ESS score > 10 indicated abnormal daytime somnolence, PSQI score > 5 bad sleep quality. We analyzed correlation with disease severity and characteristics, Hospital Anxiety and Depression Scale (HADS), Modified Fatigue Impact Scale (MFIS), Body Mass Index, drug use.

**Results:**

ESS and PSQI questionnaires were filled by 257 and 253 CMT patients, respectively, and 58 controls. Median PSQI score was higher in CMT patients than controls (6 vs 4, *p* = 0.006), with no difference for ESS score. Abnormal somnolence and poor sleep quality occurred in 23% and 56% of patients; such patients had more frequently anxiety/depression, abnormal fatigue, and positive sensory symptoms than those with normal ESS/PSQI. Moreover, patients with PSQI score > 5 had more severe disease (median CMT Examination Score, CMTES, 8 vs 6, *p* = 0.006) and more frequent use of anxiolytic/antidepressant drugs (29% vs 7%, *p* < 0.001).

**Conclusions:**

Bad sleep quality and daytime sleepiness are frequent in CMT and correlated with anxiety, depression and fatigue, confirming that different components affect sleep. Sleep disorders, such as sleep apnea and restless leg syndrome, not specifically investigated here, are other factors known to impact on sleep quality and somnolence. CMT patients’ management must include sleep behavior assessment and evaluation of its correlated factors, including general distress and fatigue.

**Supplementary Information:**

The online version contains supplementary material available at 10.1007/s00415-023-11911-y.

## Introduction

Charcot–Marie–Tooth disease (CMT) collects a genetically heterogeneous group of hereditary peripheral neuropathies with a common phenotype usually characterized by predominantly distal sensory and motor involvement, with muscle weakness and atrophy, frequent sensory loss, and foot deformities [1].

Sleep abnormalities significantly impact on quality of life and may have different causes. A few reports focusing on sleep abnormalities in CMT patients have been published [2–6], but data about daytime sleepiness and sleep quality in CMT patients are scanty.

Boentert and colleagues performed a web-based survey in 227 CMT patients and they found excessive daytime sleepiness in 31.7% of them as assessed with the Epworth Sleepiness Scale (ESS), while 79.3% of subjects were bad sleepers according to the Pittsburgh Sleep Quality Index (PSQI) [4]. Souza and coauthors administered the same scales to 16 CMT patients from a four-generation family with axonal CMT2 and found that 31% of them had abnormal sleepiness and 75% reported poor sleep quality [5].

Sleep behavior has been poorly investigated in other neuromuscular disorders with the exception of Myotonic Dystrophy (MD), where hypersomnolence was reported by 39% of 36 MD patients by Rubinsztein and colleagues, but by none of the 13 CMT patients used as diseased controls, as assessed by The Maudsley Hospital Sleep Questionnaire, which analyses sleep patterns and includes the ESS [7]. Philips et al., by employing the ESS and a sleep diary, found that both MD (*n* = 35) and CMT (*n* = 13) patients had significantly more daytime somnolence and reduced sleep quality as compared to healthy controls, as well as a higher rate of mood disorders [8]. Tielemann and coauthors reported the same percentage (44.8%) of hypersomnolence (abnormal ESS) and poor sleep quality (abnormal PSQI) in 29 MD patients [9].

ESS [10], assessing daytime sleepiness, and PSQI [11], evaluating sleep quality, are questionnaires widely used across different clinical populations, not only for the assessment of sleep disorders, but also to investigate sleep disruption in neurological disorders, such as epilepsy and Parkinson disease [12–14].

Previous studies on CMT were conducted on limited numbers of patients (with only one large series investigated) and correlations with clinical scales and features were only partial. Therefore, we aimed at verifying whether daytime sleepiness and bad sleep quality are really an issue in CMT and if so which are the factors that may be related to such abnormalities, including disease severity and CMT type. Specifically, we assessed sleep behavior using the ESS and PSQI questionnaires in a large series of CMT patients and analyzed correlations with disease severity, clinical characteristics, general distress, fatigue, body weight, and drug use.

## Methods

CMT patients were recruited among those adhering to the Italian National CMT registry, as recently described [15–18]. All the patients recruited in the registry were invited by an email to participate in the study and complete the online questionnaires (10 overall): 257 out of 850 filled the sleep questionnaires. The Registry collects a minimal data set of information and values of the CMT Neuropathy Score/Examination Score, CMTES/CMTNS) [19]. Data on Body Mass Index (BMI) and co-occurrence of diabetes are also gathered. Healthy controls were recruited among patients’ friends and relatives, matched as much as possible for gender and age.

This study was approved by the Ethics Committees of the Fondazione IRCCS Istituto Neurologico Carlo Besta, Milan (no. 52/2016 Date: April 2, 2014) and of the other participating centers, and has, therefore, been performed in accordance with the ethical standards laid down in the 1964 Declaration of Helsinki and its later amendments.

Recruitment was conducted before the COVID-19 pandemic and lasted 3 years (2015–2017).

Both patients and controls were asked to complete online the Italian validated versions of the ESS and PSQI [10, 11]. A written informed consent was signed by all participants to the Registry, while an online informed consent was signed by those who completed the online questionnaires.

The ESS is a self-assessed questionnaire composed of eight items investigating somnolence. Each item is rated by a four-point (0–3) system (range 0–24, were 0 is absence of somnolence and 24 the worst score) [10]. A total score > 10 indicates abnormal somnolence, according to Johns et al. [20].

The PSQI is a self-assessed questionnaire which retrospectively measures sleep quality over a 1-month period. It contains nineteen items measuring seven different aspects of sleep (subjective sleep quality, sleep latency, sleep duration, habitual sleep efficiency, sleep disturbances, use of sleep medications, and daytime dysfunction). Each item is rated by a four-point (0–3) system, while the global PSQI score is calculated by totaling the seven component scores with a range from 0 to 21, where lowest scores denote good sleep quality [11, 21]. A total score > 5 indicates bad sleep quality [11, 21].

Both patients and controls were asked to fill in also questionnaires concerning the presence of anxiety, depression, and general distress (Hospital Anxiety and Depression Scale, HADS, Italian version) [22] and the perception of fatigue (Modified Fatigue Impact Scale, MFIS, Italian version) [23] to explore the relation of neuropsychiatric status and fatigue with sleepiness and sleep quality. As previously described in our recent researches [15, 16], we used as cutoff scores ≥ 11 for both the HADS-A and HADS-D to define the presence of anxiety and depression, respectively, and ≥ 22 for the whole scale (HADS-T) to indicate the presence of general distress [24], while the cutoff > 38 for the MFIS scale was adopted to indicate the presence of fatigue [25]. The MFIS scale is divided into physical (PH), cognitive (C), and psychosocial (PS) subscales [23].

The questionnaire investigated also the use of antidepressants/anxiolytics and analgesics/anti-inflammatories. We considered medication users the patients taking the drugs ≥ once per week.

### Statistical analysis

Participant characteristics at baseline were described in terms of absolute numbers and percentages for categorical data and means with standard deviations (SDs) and medians for continuous data. The *t* test, Mann–Whitney *U* test, Fisher’s exact test, and Spearman’s Rank-Order Correlation, were used to analyze associations between ESS and PSQI scores and type of participants (CMT patients vs controls), gender, age, disease duration, disease severity (CMTES), walking ability and/or use of orthotics, hand disability, sensory symptoms, BMI, HADS scores, MFIS score, and medication use (antidepressant/anxiolytic and analgesic/anti-inflammatory drugs). Bonferroni correction was performed, as appropriate. Throughout the statistical analysis, the significance level was set at 0.05 (significant: < 0.05).

## Results

The ESS and PSQI questionnaires were filled in by 257 and 253 CMT patients, respectively, and by 58 controls. Demographic and clinical data, ESS and PSQI scores of CMT patients and controls are reported in Table 1. There was no significant difference in age and gender distribution between patients and controls for both ESS and PSQI score. Among the 257 CMT patients, 119 (46.3%) had a diagnosis of CMT1A; the other most frequent CMT types were CMTX1 (*n* = 21, 8.2%), CMT1B (*n* = 19, 7.4%), CMT2I/CMT2J (*n* = 14, 5.4%), CMT2A (*n* = 8, 3.1%), CMT4C (*n* = 5, 2.0%); 2 CMT1A, 1 CMT2A, and 1 CMT1B patient did not fill the PSQI questionnaire.

**Table 1:** Demography, disease severity, ESS and PSQI scores of CMT patients and healthy controls

	CMT Patients			Controls		
	Females* (*n* = 141)	Males (*n* = 116)	Total* (*n* = 257)	Females (*n* = 26)	Males (*n* = 32)	Total (*n* = 58)
Age (mean ± SD, median)	46.6 ± 12.2, 47	47.3 ± 13.8, 50	46.9 ± 12.9, 48	46.7 ± 12.4, 49	43.9 ± 12.6, 44	45.2 ± 12.5, 46
CMTES (mean ± SD, median)	8.1 ± 5.2, 7	8.3 ± 5.1, 8	8.2 ± 5.2, 8	–	–	–
ESS Score (mean ± SD, median)	7.7 ± 4.5, 7	6.8 ± 4.0, 7	7.3 ± 4.3, 7	6.7 ± 4.8, 6.5	6.5 ± 3.9, 5.5	6.6 ± 4.3, 6
ESS Score > 10, *n* (%)	42 (16%)	18 (7%)	60 (23%)	5 (9%)	7 (12%)	12 (21%)
PSQI score*^a,b^ (mean ± SD, median)	7.3 ± 3.7, 7^b^	5.6 ± 3.2, 5 ^b^	6.5 ± 3.5, 6^a^	5.8 ± 3.6, 5	4.9 ± 2.8, 4	5.3 ± 3.2, 4^a^
PSQI Score > 5*^c^, *n* (%)	86 (63%)	55 (47%)	141 (56%)^c^	12 (46%)	9 (28%)	21 (36%)^c^
Component 5 PSQI*^d^ (mean ± SD, median)	–	–	1.6 ± 0.6; 2^d^	–	–	1.2 ± 0.7; 1^d^

*CMT* Charcot–Marie–Tooth disease, *CMTES* Charcot–Marie–Tooth Examination Score, *ESS* Epworth Sleepiness Scales, *PSQI* Pittsburgh Sleep Quality Index, *SD* standard deviation

Note: there were significant differences between CMT and controls neither for ESS scores (*p* = 0.252, Mann–Whitney *U* test) nor for ESS > 10 (*p* = 0.732, Fisher’s exact test)

*Four females did not complete the PSQI questionnaire

^a^PSQI score was significantly higher in CMT patients than in controls (p = 0.006, Mann–Whitney *U* test) and ^b^In CMT females than in males (*p* < 0.001, Mann–Whitney *U* test)

^c^Abnormal sleep quality (PSQI > 5) was more frequently reported by CMT patients as compared to controls (*p* = 0.008, Fisher’s exact test)

^d^Component 5 of PSQI score was significantly higher in CMT patients than in controls (*p* < 0.001, Mann–Whitney *U* test)

### ESS scores

The mean and median values of ESS scores of CMT patients did not significantly differ either from controls (*p* = 0.252), or between CMT patients’ genders (*p* = 0.081) (Table 1).

Among CMT patients, 23% (60/257) had abnormal levels of daytime somnolence (ESS > 10), a rate similar to controls (21%, *p* = 0.732) (Table 1). Patients with abnormal daytime somnolence were more frequently females (70% vs 50%, *p* = 0.007), and more often reported hand disability (difficulties with buttons 68% vs 49%, *p* = 0.008) and positive sensory symptoms (40% vs 28%, *p* = 0.009) (Table 2).

**Table 2:** Comparisons between CMT patients with normal and abnormal ESS/PSQI scores for clinical characteristics, HADS, MFIS, BMI, and drug consumption

	Daytime sleepiness			Sleep quality		
	ESS Score ≤ 10 (*n* = 197)	ESS Score > 10 (*n* = 60)	*p* value*	PSQI Score ≤ 5 (*n* = 112)	PSQI Score > 5 (*n* = 141)	*p* value*
Gender	**Females 99 (50%)** **Males 98 (50%)**	**Females 42 (70%)** **Males 18 (30%)**	**0.007**	**Females 51/(46%)** **Males 61/(54%)**	**Females 86 (61%)** **Males 55 (39%)**	**0.016**
Age (mean ± SD, median)	46.9 ± 13.2, 48	46.9 ± 11.9, 47	n.s	**44.3 ± 12.7, 44**	**49.1 ± 12.9, 51**	**0.002**
Disease duration (mean ± SD, median)	24.4 ± 14.9, 24	24.2 ± 14.4, 23	n.s	23.7 ± 14.1, 23	24.9 ± 15.3, 23	n.s
CMTES (mean ± SD, median)	8.1 ± 5.1, 8	8.5 ± 5.2, 8	n.s	**7.4 ± 5.1, 6**	**8.9 ± 5.2, 8**	**0.006**
Walking difficulties, *n* (%)	145 (74%)	48 (80%)	n.s	78 (70%)	112 (79%)	n.s
Ankle foot orthotics, *n* (%)	31 (22%)	9 (15%)	n.s	12 (11%)	27 (19%)	n.s
Walking support need, *n* (%)	22 (11%)	10 (17%)	n.s	8 (7%)	24 (17%)	n.s
Difficulties with buttons, *n* (%)	**96 (49%)**	**41 (68%)**	**0.008**	61 (54%)	89 (63%)	n.s
Positive sensory symptoms, *n* (%)	**55 (28%)**	**24 (40%)**	**0.009**	**32 (29%)**	**63 (45%)**	**0.009**
Anxiety HADS A Score (mean ± SD, median)	**6.4 ± 4.7, 5**	**8.8 ± 4.7, 9**	** < 0.001**	**5.0 ± 4.0, 4**	**8.5 ± 4.9, 8**	** < 0.001**
Depression HADS D Score (mean ± SD, median)	**4.2 ± 3.7, 3**	**5.9 ± 4.5, 6**	**0.007**	**2.9 ± 2.7, 2**	**6.0 ± 4.4, 5**	** < 0.001**
General distress HADS T Score (mean ± SD, median)	**10.6 ± 7.9, 8**	**14.8 ± 8.3, 14**	** < 0.001**	**7.9 ± 6.2, 6**	**14.4 ± 8.4, 14**	** < 0.001**
MFIS (mean ± SD, median)	**28.8 ± 17.5, 30**	**42.6 ± 17.4, 41**	** < 0.001**	**22.3 ± 15.7, 20**	**40.0 ± 16.6, 40**	** < 0.001**
Anxiolytics/antidepressants use, n (%)	43/193 (22%)	13/46 (28%)	n.s	**7/107 (7%)**	**37/125 (29%)**	** < 0.001**
Analgesics/anti-inflammatory use, *n* (%)	15/70 (21%)	41/169 (24%)	n.s	73/109 (67%)	94/127 (74%)	n.s
BMI (mean ± SD, median)	24.4 ± 4.1, 23	25.4 ± 5.1, 24	n.s	28.8 ± 17.5, 30	28.8 ± 17.5, 30	n.s

*p* values for significant differences are reported in bold

*A* anxiety, *BMI* Body Mass Index, *CMT* Charcot–Marie–Tooth disease, *CMTES* Charcot–Marie–Tooth Examination Score, *D* depression, *ESS* Epworth Sleepiness Scale, *F* females, *HADS* Hospital Anxiety and Depression Scale, *M* males, *MFIS* Modified Fatigue Impact Scale, *n.s.* not significant, *PSQI* Pittsburgh Sleep Quality Index, *SD* standard deviation, *T* total

*p value calculated using the *t* test, Mann–Whitney *U* test or the Fisher’s exact test as appropriate

As compared to CMT subjects with normal daytime somnolence (ESS score ≤ 10), they also more frequently reported anxiety/depression/general distress according to the HADS (median HADS-A 9 vs 5, median HADS-D 6 vs 3, median HADS-T 14 vs 8, *p* < 0.001, *p* = 0.007 and *p* < 0.001, respectively) and abnormal fatigue, according to the MFIS (median 41 vs 30, *p* < 0.001) (Table 2).

On the other hand, we found no difference for age, disease duration, disease severity (as assessed by the CMTES), lower limb disability, BMI, use of antidepressants/anxiolytics, and anti-inflammatory/analgesic drugs consumption (Table 2). There was no difference in daytime sleepiness according to CMT type (median ESS score: CMT1A = 7 vs other CMT types = 7, *p* = 0.260).

We then examined the correlations between daytime sleepiness scores and disease severity (CMTES), general distress, fatigue, BMI, sleep quality (Supplementary Table 1), and diabetes presence (*n* = 10). We found a slight correlation between somnolence and anxiety/depression/general distress (HADS-A, HADS-D, HADS-T, *r* = 0.36, *r* = 0.32, *r* = 0.37, respectively), while a stronger correlation was found between somnolence and general/physical/cognitive fatigue (*r* = 0.40, *r* = 0.49, *r* = 0.49, respectively).

### PSQI scores

Median PSQI scores were significantly higher in CMT patients in comparison with controls (*p* = 0.006) (Table 1), and abnormal sleep quality (PSQI > 5) was more frequently reported by CMT patients (56%, 141/253) than controls (36%, 21/58, *p* = 0.008). We found no significant difference between friends and relatives in the control group, suggesting that the same environmental factors and genetic background between patients and relatives did not influence the analyses.

As compared to CMT subjects with normal sleep quality (PSQI ≤ 5), patients with abnormal sleep quality were more frequently females (*p* = 0.016), were significantly older (mean 49.1 vs 44.3 years of age, *p* = 0.002), had more severe disease as assessed by the CMTES (median 8 vs 6, *p* = 0.006), and more frequently reported abnormal fatigue as defined by MFIS (median 40 vs 20, *p* < 0.001), anxiety/depression/general distress as defined by HADS (median value: HADS-A 8 vs 4, HADS-D 5 vs 2, HADS-T 14 vs 6, respectively, *p* < 0.001 for all comparisons), positive sensory symptoms (45% vs 29%, *p* = 0.009) and anxiolytic/antidepressant drugs consumption (29% vs 7%, *p* < 0.001) (Table 2).

We found no difference in PSQI scores for disease duration, use of orthotics, walking and hand disability, CMT type (Supplementary Table 2), BMI, and co-occurrence of diabetes.

Notably, the component of the PSQI score, where there was a significant difference between CMT patients and healthy controls (median 2 vs 1, *p* < 0.001) was the fifth component, which investigates sleep disturbances. Component five of PSQI score is made up of ten sub-items, each analyzing different symptoms: cannot get to sleep within 30 min, wake up in the middle of the night or early morning, have to get up to use the bathroom, cannot breathe comfortably, cough or snore loudly, feel too cold or too hot, have bad dreams, have pain, other reasons. CMT patients more frequently reported that they could not comfortably breathe (32% vs 10%, *p* = 0.010), felt too cold (49% vs 22%, *p* = 0.002) or too hot (59% vs 20%, *p* = 0.007), and had nocturnal pain (70% vs 43%, *p* = 0.003) (Table 3).

**Table 3:** Comparison of PSQI component 5 subitems’ results in CMT patients and healthy controls

	CMT patients (*n* = 253)	Controls (*n* = 58)	*p* value
1. Cannot get to sleep within 30 min, *n* (%)	154 (61%)	34 (59%)	n.s
2. Wake up in the middle of the night or early morning, *n* (%)	221 (87%)	43 (74%)	n.s
3. Have to get up to use the bathroom, *n* (%)	184 (73%)	39 (67%)	n.s
4. Cannot breathe comfortably, *n* (%)	**80 (32%)**	**6 (10%)**	**0.010**
5. Cough or snore loudly, *n* (%)	126 (50%)	30 (52%)	n.s
6. Feel too cold, *n* (%)	**125 (49%)**	**13 (22%)**	**0.002**
7. Feel too hot, *n* (%)	**150 (59%)**	**20 (34%)**	**0.007**
8. Have bad dreams, *n* (%)	136 (54%)	25 (43%)	n.s
9. Have pain, *n* (%)	**177 (70%)**	**25 (43%)**	**0.003**
10. Other reasons, *n* (%)	48 (19%)	8 (12%)	n.s
Cramps, *n* (%)	7 (3%)	0	n.s
Anxiety/stress, *n* (%)	34 (13%)	6 (10%)	n.s
Paresthesias, *n* (%)	7 (3%)	1 (2%)	n.s

P values for significant difference are reported in bold

*CMT* Charcot–Marie–Tooth disease, *ns* not significant

*p value calculated using the Fisher’s exact test. *p* values were adjusted using the Bonferroni correction

As compared to males, female patients more frequently complained of feeling too cold (59% vs 38%, *p* = 0.001) and of nocturnal pain (80% vs 58%, *p* < 0.001).

No difference was found for the other PSQI components between patients and control.

We also examined the correlations between sleep quality and disease severity (CMTES), general distress, fatigue, sleepiness, BMI (Supplementary Table 1), and diabetes. We found a moderate correlation with PSQI for age, anxiety/depression/general distress (HADS-A, HADS-D, HADS-T, *r* = 0.41, *r* = 0.46, *r* = 0.47, respectively), physical/cognitive/general fatigue (MFIS-PH, MFIS-C, MFIS-T, *r* = 0.53, *r* = 0.44, *r* = 0.55, respectively), and daytime somnolence.

## Discussion

We investigated sleep in a large series of 257 CMT patients and 58 healthy controls using the ESS and PSQI, two questionnaires widely used to investigate daytime sleepiness and sleep quality, respectively. There are only a few studies [2–6] investigating sleep in CMT and only one involved a large series of patients (*n* = 227) [4]. We found excessive daytime somnolence (ESS > 10) in 23% of CMT patients, a percentage comparable to controls and slightly lower than that found by Boentert and colleagues (31.7%) and Souza and coauthors (31% of 16 patients) [4–6]. Bad sleep quality (PSQI > 5) was reported by 56% of our patients, a rate higher than controls, but lower than previously reported (75.9–85.2%) [4, 5, 26]. Our CMT patients showed less daytime sleepiness in comparison with subjects with myotonic dystrophy (mean ESS score 7.3 vs 9.6, reported by Tieleman et al., and 11 by Phillips et al.), while sleep quality was comparable (mean PSQI score 6.2 vs 6.3 found by Tieleman et al.), and the percentage of bad sleepers was similar (56% vs 45% in MD patients) [8, 9].

Daytime somnolence, therefore, is an issue for almost one quarter of CMT patients and sleep quality is bad for most of them. However, which are the causes of these findings, and which are the correlations with the disease characteristics?

Sleepiness does not seem to be related to overall disease severity, though hand disability and positive sensory symptoms including pain were more frequent among CMT patients with somnolence. Female gender, anxiety and depression, and fatigue, but not age and BMI, were factors associated with daytime sleepiness.

Bad sleep quality was associated with overall disease severity and positive sensory symptoms, older age, female gender, presence of fatigue, anxiety and depression, and use of anxiolytics/antidepressants. The PSQI component 5 was the most abnormal one in CMT subjects and its analysis revealed that, with respect to controls, they more frequently could not breathe comfortably (32%), felt too cold (49%) or too hot (59%), and had nocturnal pain (70%); women reported more frequently than males feeling too cold and nocturnal pain. Notably, sleepiness and bad sleep quality were independent from CMT type.

These findings suggest that sleep abnormalities are partly related to the CMT disease itself, particularly because of pain and subjective sensory abnormalities, partly to mood disorders and fatigue, which are also frequent in CMT and linked to the condition of chronic disease as previously shown by us [16, 17] and others [4, 5, 7, 26], and likely also by the occurrence of specific sleep disorders in the CMT population as reported in previous studies [4, 5, 7, 26].

Indeed, an increased prevalence of sleep apneas, Restless Leg Syndrome (RLS), Periodic Limb Movements in Sleep (PMLS), have been reported in CMT [26]. Nocturnal cramps are another reported reason for bad quality of sleep [6].

Gemignani and coauthors were the first to report a high rate of RLS in patients with the axonal CMT2 type [27]. The study by Boentert et al. confirmed an increased prevalence of RLS in CMT (18.1%) and found that its severity was correlated with worse sleep quality [4].

Dematteis and colleagues first reported the occurrence of sleep apneas in 11 members of a large family with CMT1 [2]. In a first case–control study on 12 CMT patients and 24 disease controls, Dziewas et al. found at polysomnography an increased prevalence of obstructive sleep apneas (OSA) in CMT subjects, with a mean apnea–hypopnea index (AHI) of 10.5 (normal below 10, disease controls = 1.5) [3]. In a second larger case–control study on 61 CMT subjects and 61 matched insomniac controls, the same group confirmed an increased rate of OSA in the CMT group (37.7% vs 4.9% in controls) and a mean AHI of 9.1 (vs 1.2 in controls, normal ≤ 5), positively correlated with age and disease severity [26]. They also found a higher prevalence of RLS (40.9% vs 16.4% in controls) and PMLS (41% vs 22.9%) [26]. Sleep apneas in CMT are mainly obstructive in nature and believed to be related to involvement of pharyngeal nerves increasing the collapsibility of the upper airways; vocal cord palsy and diaphragmatic weakness are rare predisposing conditions [4, 26, 28].

An important limitation of our study is that we did not investigate the presence of OSA, RLS and PLMS, mainly because the patients had already to fill many questionnaires online (*n* = 11) and we feared that others would have reduced responders’ rate. Therefore, we cannot draw conclusions about the role of sleep disorders on sleepiness and bad sleep quality in our series.

In summary, we found a higher rate of bad sleepers among CMT patients, while daytime sleepiness was not increased but still was reported by 23% of the patients. We propose that there are different contributing factors, including disease severity, sensory abnormalities, nocturnal pain and cramps, mood disorders, fatigue, and, based on the literature, sleep disorders, such as OSA and RLS. Therefore, CMT patients’ management must include sleep abnormalities' assessment and evaluation of their different associated factors. Proper management of such factors may alleviate disease burden, ameliorate sleep quality, and reduce daytime sleepiness. A large multicenter international study is warranted to firmly establish a correlation between specific sleep disorders and CMT.

### Supplementary Information

Below is the link to the electronic supplementary material.

Supplementary file1 (DOCX 24 KB)

## Data Availability

Data relevant to the study are included in the article or uploaded as online supplemental information. Data supporting study results are deposited in an ad hoc repository and are available from the Principal Investigator (DP) to be shared anonymously on request from any qualified investigator.
