# Correction to: Daytime sleepiness and sleep quality in Charcot–Marie–Tooth disease

**DOI:** 10.1007/s00415-023-11989-4

**Published:** 2023-09-21

**Authors:** Marta Bellofatto, Luca Gentile, Alessandro Bertini, Irene Tramacere, Fiore Manganelli, Gian Maria Fabrizi, Angelo Schenone, Lucio Santoro, Tiziana Cavallaro, Marina Grandis, Stefano C. Previtali, Marina Scarlato, Isabella Allegri, Luca Padua, Costanza Pazzaglia, Flavio Villani, Eleonora Cavalca, Paola Saveri, Aldo Quattrone, Paola Valentino, Stefano Tozza, Massimo Russo, Anna Mazzeo, Giuseppe Vita, Sylvie Piacentini, Giuseppe Didato, Chiara Pisciotta, Davide Pareyson, for the Italian C. M. T. Network, Giulia Schirinzi, Maria Montesano, Sara Nuzzo, Francesca Oggiano, Daniela Calabrese, Chiara Gemelli, Yuri Falzone, Emanuele Spina, Maria Longo, Giuseppe Occhipinti, Giacomo Iabichella, Stefania Barone

**Affiliations:** 1 SC Malattie Neurologiche Rare, Dipartimento di Neuroscienze Cliniche, Fondazione IRCCS Istituto Neurologico Carlo Besta, Via Celoria 11, 20133 Milan, Italy; 2 Unità di Neurologia e Malattie Neuromuscolari, Dipartimento di Medicina Clinica e Sperimentale, Università di Messina, 98124 Messina, Italy; 3 Dipartimento Gestionale di Ricerca e Sviluppo Clinico, Direzione Scientifica, Fondazione IRCCS Istituto Neurologico Carlo Besta, 20133 Milan, Italy; 4 Dipartimento di Neuroscienze, Scienze Riproduttive ed Odontostomatologiche, Università Federico II di Napoli, 80131 Naples, Italy; 5 Dipartimento di Neuroscienze, Biomedicina e Movimento, Università di Verona, 37126 Verona, Italy; 6 Dipartimento di Neuroscienze, Riabilitazione, Oftalmologia, Genetica e Scienze Materno-Infantili, Università di Genova, 16132 Genoa, Italy; 7 IRCCS Ospedale Policlinico San Martino, 16132 Genoa, Italy; 8 INSPE and Division of Neuroscience, IRCCS Ospedale San Raffaele, 20132 Milan, Italy; 9 U.O. Neurologia, A.O. di Parma, 42126 Parma, Italy; 10 Università Cattolica del Sacro Cuore, 00168 Rome, Italy; 11 Fondazione Policlinico Universitario A. Gemelli IRCCS, 00168 Rome, Italy; 12 Unità di U.O. Neurofisiopatologia, IRCCS Ospedale Policlinico San Martino, 16132 Genoa, Italy; 13 Dipartimento di Scienze Mediche, Università Magna Grecia, 88100 Catanzaro, Italy; 14 Unità di Neuropsicologia, Dipartimento di Neuroscienze Cliniche, Fondazione IRCCS Istituto Neurologico Carlo Besta di Milano, 20133 Milan, Italy; 15 Unità di Epilettologia Clinica e Sperimentale, Dipartimento di Neuroscienze Cliniche, Fondazione IRCCS Istituto Neurologico Carlo Besta, 20133 Milan, Italy

**Correction to: Journal of Neurology** 10.1007/s00415-023-11911-y

The article Daytime sleepiness and sleep quality in Charcot–Marie–Tooth disease, written by Marta Bellofatto, Luca Gentile, Alessandro Bertini, Irene Tramacere, Fiore Manganelli, Gian Maria Fabrizi, Angelo Schenone, Lucio Santoro, Tiziana Cavallaro, Marina Grandis, Stefano C. Previtali, Marina Scarlato, Isabella Allegri, Luca Padua, Costanza Pazzaglia, Flavio Villani, Eleonora Cavalca, Paola Saveri, Aldo Quattrone, Paola Valentino, Stefano Tozza, Massimo Russo, Anna Mazzeo, Giuseppe Vita, Sylvie Piacentini, Giuseppe Didato, Chiara Pisciotta, Davide Pareyson and for the Italian C. M. T. Network was originally published electronically on the publisher’s internet portal on 04 August 2023 without open access. With the author(s)’ decision to opt for Open Choice the copyright of the article changed on 19 August 2023 to © The Authors 2023 and this article is licensed under a Creative Commons Attribution 4.0 International License, which permits use, sharing, adaptation, distribution and reproduction in any medium or format, as long as you give appropriate credit to the original author(s) and the source, provide a link to the Creative Commons licence, and indicate if changes were made. The images or other third party material in this article are included in the article’s Creative Commons licence, unless indicated otherwise in a credit line to the material. If material is not included in the article’s Creative Commons licence and your intended use is not permitted by statutory regulation or exceeds the permitted use, you will need to obtain permission directly from the copyright holder. To view a copy of this licence, visit http://creativecommons.org/licenses/by/4.0/.

The original article has been corrected.

