# Supplementary material for: Daytime sleepiness and sleep quality in Charcot–Marie–Tooth disease
Source: J Neurol. 2023 Aug 4;270(11):5561–8. doi: 10.1007/s00415-023-11911-y (PMC10576706; doi:10.1007/s00415-023-11911-y)
Supplement: Supplementary file 1 — Supplementary file1 (DOCX 24 KB) [file 415_2023_11911_MOESM1_ESM.docx]

**Supplementary Table 1. Correlations between ESS and PSQI scores and clinical characteristics, HADS, MFIS, and BMI.**

|  | **Daytime Somnolence**  **ESS Score** | **Sleep Quality**  **PSQI Score** |
| --- | --- | --- |
| **Age** | r: -0.02  p=n.s. | **r: 0.19**  **p <0.002** |
| **CMTES** | r: 0.04  p=0.050 | **r: 0.22**  **p<0.001** |
| **Disease duration** | r: -0.05  p=n.s. | r: 0.41  p=n.s. |
| **Anxiety**  **HADS-A Score** | **r: 0.36**  **p<0.001** | **r: 0.41**  **p<0.001** |
| **Depression**  **HADS-D Score** | **r: 0.32**  **p<0.001** | **r: 0.46**  **p<0.001** |
| **General Distress**  **HADS-T Score** | **r: 0.37**  **p<0.001** | **r: 0.47**  **p<0.001** |
| **General Fatigue**  **MFIS-T Score** | **r: 0.49**  **p<0.001** | **r: 0.55**  **p<0.001** |
| **Physical Score**  **MFIS-PH Score** | **r: 0.40**  **p<0.001** | **r: 0.53**  **p<0.001** |
| **Cognitive Score**  **MFIS-C Score** | **r: 0.49**  **p<0.001** | **r: 0.44**  **p<0.001** |
| **Daytime Somnolence**  **ESS Score** | **--------** | **r: 0.32**  **p<0.001** |
| **BMI** | r: 0.06  p=n.s. | r: 0.11  p=n.s. |

A=Anxiety; BMI=Body Mass Index; C=Cognitive; CMTES=Charcot-Marie-Tooth Examination Score; D=Depression; ESS=Epworth Sleepiness Scale; HADS=Hospital Anxiety and Depression Scale; MFIS=Modified Fatigue Impact Scale; n.s.= not significant; PH=physical; PSQI=Pittsburgh Sleep Quality Index; T=Total.

Significant P values are reported in bold. *P value calculated using the Spearman’s Rank-Order Correlation.

**Supplementary Table 2.** Comparison between CMT1A, other CMT subtypes and controls for PSQI and bad sleep quality.

|  | **CMT1A** (117) | **Other CMT**  **subtypes** (140) | **Controls** (58) |
| --- | --- | --- | --- |
| **PSQI score**  (Mean ± SD, median) | 6.5 ± 3.6, 6  p=0.002* | 6.4 ± 3.4, 6  p=0.008* | 5.3 ± 3.2, 4 |
| **PSQI>5**  n (%) | 64 (55%)  p=0.024* | 80 (57%)  p=0.008* | 21 (36%) |

**Legend.** PSQI=Pittsburgh Sleep Quality Index; SD=Standard Deviation.

*P values calculated versus controls.

There was no significant difference between CMT1A and other CMT subtypes.
